# Vital Statistics of the Medical Profession

**Published:** 1852-05

**Authors:** 


*Vital Statistics of the Medical Profession.*—It appears from the annual report, read by the Secretary of the Medical Society of the first district of Paris, that out of the 80 members who have been connected with the Society for the last seven years, three only have died. This first district (one of the cleanest and wealthiest in Paris,) seems to be favored in a sanitary point of view. According to the returns, one inhabitant, in that district, dies out of 58.66; whilst over the whole of France there is one death in 39-7 inhabitants. The above statements would seem rather favorable to the medieal profession, but the following table, published by Casper of Berlin, is not so encouraging. Out of a hundred persons engaged in different callings, the following have lived beyond 70 years :—Divines, 42; agriculturists, 40; merchants, and persons holding the higher offices, 35; soldiers, and people holding situations of an humble description, 32; barristers, 29; cultivators of any of the fine arts, 27; professors and teachers, 27; and *medical men* 24.—*Ibid.*

